# Evaluation of a Functional Single Nucleotide Polymorphism of the SARS-CoV-2 Receptor *ACE2* That Is Potentially Involved in Long COVID

**DOI:** 10.3389/fgene.2022.931562

**Published:** 2022-07-18

**Authors:** Yu-Si Luo, Lei Luo, Wei Li, Yan Chen, Guo-Feng Wu, Fang Chen, Hu-Yan Shen, Hong-Man Li, Ming-Yang Guo, Sha Yin, Ke Zhang, Zhong-Shan Cheng

**Affiliations:** ^1^ Department of Emergency, The Affiliated Hospital of Guizhou Medical University, Guiyang, China; ^2^ The Key and Characteristic Laboratory of Modern Pathogenicity Biology, School of Basic Medical Sciences, Guizhou Medical University, Guiyang, China; ^3^ Good Clinical Practice Center, Guizhou Provincial People’s Hospital, Guiyang, China; ^4^ Department of Cardiovascular Medicine, The Affiliated Hospital of Guizhou Medical University, Guiyang, China; ^5^ The High Efficacy Application of Natural Medicinal Resources Engineering Center of Guizhou Province, School of Pharmaceutical Sciences, Guizhou Medical University, Guiyang, China; ^6^ Department of Hypertension, The Affiliated Hospital of Guizhou Medical University, Guiyang, China; ^7^ Center for Applied Bioinformatics, St. Jude Children’s Research Hospital, Memphis, TN, United States

**Keywords:** SARS-CoV-2, COVID-19, long COVID, ACE2, rs2106809

## Abstract

Since the occurrence of severe acute respiratory syndrome coronavirus 2 (SARS-CoV-2) in December 2019, SARS-CoV-2 has led to a global coronavirus disease 2019 (COVID-19) pandemic. A better understanding of the SARS-CoV-2 receptor *ACE2* at the genetic level would help combat COVID-19, particularly for long COVID. We performed a genetic analysis of *ACE2* and searched for its common potential single nucleotide polymorphisms (SNPs) with minor allele frequency >0.05 in both European and Chinese populations that would contribute to *ACE2* gene expression variation. We thought that the variation of the *ACE2* expression would be an important biological feature that would strongly affect COVID-19 symptoms, such as “brain fog”, which is highlighted by the fact that ACE2 acts as a major cellular receptor for SARS-CoV-2 attachment and is highly expressed in brain tissues. Based on the human GTEx gene expression database, we found rs2106809 exhibited a significant correlation with the *ACE2* expression among multiple brain and artery tissues. This expression correlation was replicated in an independent European brain eQTL database, Braineac. rs2106809*G also displays significantly higher frequency in Asian populations than in Europeans and displays a protective effect (*p* = 0.047) against COVID-19 hospitalization when comparing hospitalized COVID-19 cases with non-hospitalized COVID-19 or SARS-CoV-2 test-negative samples with European ancestry from the UK Biobank. Furthermore, we experimentally demonstrated that rs2106809*G could upregulate the transcriptional activity of *ACE2*. Therefore, integrative analysis and functional experiment strongly support that *ACE2* SNP rs2106809 is a functional brain eQTL and its potential involvement in long COVID, which warrants further investigation.

## Introduction

*ACE2* encodes the protein angiotensin-converting enzyme (ACE) 2, which is a receptor of SARS-CoV-2 ^[1-3]^. Before ACE2 was identified as the SARS-CoV-2 receptor, it was well known as a negative regulator of the renin–angiotensin system (RAS). The functions of ACE2 in the RAS are to hydrolyze angiotensin (Ang) I into Ang (1–9) and to directly cleave Ang II, a powerful vasoconstrictor, to Ang (1–7) ^[4]^. Therefore, the hydrolysis of Ang I and Ang II by ACE2 strictly controls the deleterious functions of Ang II and Ang I to cardiovascular system by limiting oxidative stress and then inducing antifibrotic and vasodilatory actions ^[5]^. Recently, highly expressed Ang II-induced severe complications have been observed in COVID-19 ^[6]^. However, not only mainly expressed in the cardiovascular system, *ACE2* is also discovered to be highly decoded in brain regions such as the cerebral cortex, amygdala, and the brainstem of humans. The brainstem is a sort of key life control center for the maintenance of cardiorespiratory, cardiovascular, gastrointestinal, and neurological processes. The pons and medulla of the brainstem have the highest *ACE2* expression level ^[7]^.

Based on the sizeable scientific literature reports, ACE2 was hijacked as a functional cell receptor for virus attachment and entry by lineage B β-coronaviruses, including SARS-CoV-2 ^[1-3]^. SARS-CoV-2 was depicted as a neurotropic virus, in line with the evidence such as the capacity of SARS-CoV-2 to infect and replicate in neuro cells ^[8]^, and COVID-19 was also thought as an endothelial but not an epithelial disease^[9]^, consistent with the pieces of evidence that *ACE2* is broadly expressed on the membrane of the endothelium and pericytes which are composed of the capillaries of vascular microcirculation of all organs, including the cerebrum ^[10]^. Since SARS-CoV-2 infection reduces *ACE2* expression ^[6]^ and brainstem has the relatively higher *ACE2* expression level than other cerebral regions, the dysregulation of *ACE2* in the brainstem after SARS-CoV-2 infection might be closely associated with a novel conception: long COVID.

The British National Institute for Health and Care Excellence defines long COVID as “signs and symptoms that develop during or after an infection consistent with COVID-19, continue for more than 12 weeks, and are not explained by an alternative diagnosis” ^[11]^. The current description of signs and symptoms for long COVID includes fatigue, dyspnea, headache, anxiety, depression, cognitive disturbances (brain fog), cough, joint and chest pains, smell and taste dysfunction, and myalgia that persist for at least 4 weeks after symptom onset or hospital discharge ^[12,13]^. Further study showed 30–80% COVID-19 survivors suffered from long COVID lasting for 1–6 months ^[14]^.

Remarkably, as one potent explanation for “brain fog,” anxiety, and depression of long COVID symptoms, SARS-CoV-2 was demonstrated to infect astrocytes, one of the neuro cells distributed in the brainstem with a vigorous *ACE2* expression, and to subsequently impede the transfer of glucose and lactate from astrocytes to neurons ^[15]^. Furthermore, infection of astrocytes could lead to disruption of the blood–brain barrier; then, the systemic “cytokine storm” swarming, neuro-inflammation, and microglial activation happened ^[16]^. Such pathophysiological basis of neurological symptoms for long COVID could be led by the disrupted *ACE2* expression in brain regions, especially in the brainstem. Therefore, *ACE2* gene polymorphism might play a key role in long COVID.

As it is urgent to understand the novel neurological issue, such as “brain fog,” of long COVID, we performed an integrative genetic analysis of the SARS-CoV-2 receptor *ACE2* and prioritized a promoter SNP of *ACE2*, rs2106809, which could regulate the expression of *ACE2* in brain tissues. This study may shed light on genetics factors that are involved in long COVID.

## Methods and Materials

### *ACE2* Expression Analyses

The *ACE2* expression in diverse tissues of human body was depicted *via* the Genotype-Tissue Expression (GTEx) project. The GTEx project was established for sharing characteristic human transcriptomes within and across individuals for a wide variety of primary tissues and cell types. The expression levels of *ACE2* in all donors across 49 tissues were retrieved from GTEx V8 ^[17]^ (https://storage.googleapis.com/gtex_analysis_v8/rna_seq_data/GTEx_Analysis_2017-06-05_v8_RNASeQCv1.1.9_gene_tpm.gct.gz). Sex information for GTEx samples was obtained from the phenotype file “GTEx_Analysis_v8_Annotations_SubjectPhenotypesDS.txt” (https://storage.googleapis.com/gtex_analysis_v8/annotations/GTEx_Analysis_v8_Annotations_SubjectPhenotypesDS.txt). Tissue information for GTEx samples was collected from the file “GTEx_Analysis_v8_Annotations_SampleAttributesDS.txt” (https://storage.googleapis.com/gtex_analysis_v8/annotations/GTEx_Analysis_v8_Annotations_SampleAttributesDS.txt).

### Prioritization of the *ACE2* Promoter Single Nucleotide Polymorphism rs2106809 for the Functional Study

In order to obtain common functional SNPs regulating the *ACE2* expression in both European and Chinese populations, we downloaded all *ACE2* eQTLs from the GTEx database, where most samples are derived from European ancestry. In detail, we used *ACE2* to search for all eQTLs in the GTEx portal (https://gtexportal.org/home/); by clicking the option of “Significant Single-Tissue eQTLs for *ACE2* (ENSG00000130234.10) in all tissues,” we exported all eQTLs of *ACE2* from the GTEx portal. The total number of *ACE2* eQTLs was 215, all of which were associated with the *ACE2* expression with *p* < 0.001 across any of 49 GTEx tissues.

As our purpose was to study functional SNPs that are common between European and Chinese samples, we kept these *ACE2* eQTLs based on their minor allele frequencies (MAFs) > 0.05 in both European and Chinese populations, and the MAFs of these SNPs in the two populations were determined by PLINK2.0 ^[18]^ with the genotyping data across 503 European individuals and 103 Chinese Han in Beijing (CHB) obtained from the 1000 Genomes Project released in June 2011 ^[19]^. This filtering resulted in 140 *ACE2* eQTLs with MAF > 0.05 in both populations.

With genotyping data for these *ACE2* eQTLs of European and CHB populations from the 1000 Genomes Project, we used Haploview ^[20]^ to analyze and visualize the linkage disequilibrium (LD) pattern of these common *ACE2* eQTLs between European and Chinese samples. By evaluating the genomic region of *ACE2* in the UCSC Genome Browser, we specifically focused on *ACE2* eQTLs located in the *ACE2* promoter (chrX: 15 597 069–15 607 069; hg38), and only the SNP rs2106809 is common with MAF > 0.05 in both European and Chinese populations. Thus, this *ACE2* promoter SNP was selected for the functional study.

### Association of the *ACE2* Promoter Single Nucleotide Polymorphisms With COVID-19 Hospitalization in European Population

To determine whether the promoter SNP rs2106809 is associated with COVID-19, we evaluated the COVID-19 genome-wide association study (GWAS) summary statistics freely available at GRASP (https://grasp.nhlbi.nih.gov/Covid19GWASResults.aspx) ^[21]^. We prioritized the COVID-19 GWAS with sample sizes of cases > 1 000, controls required to be tested for SARS-CoV-2 infection, conducted in a single population without a potential ancestry effect, and COVID-19 phenotypes highly related to COVID-19 severity. We found the COVID-19 hospitalization GWAS of “Hospitalized COVID-19–positive vs. non-hospitalized COVID-19–positive or COVID-19–negative in EUR UK Biobank tested samples” met these criteria. This European COVID-19 hospitalization GWAS has 1,712 cases and 56,988 controls, the summary statistics of which were downloaded from GRASP (https://grasp.nhlbi.nih.gov/downloads/COVID19GWAS/02242021/UKBB_hsptl_EURtested_022421.txt.gz).

### Cells

The A549 (ATCC, United States, CCL-185) and HEK293T (ATCC, United States, CRL-1573) cells were, respectively, grown in T75 tissue culture flasks using DMEM (Gibco Ltd., c11995500bt) supplemented with 10% fetal bovine serum and 1% penicillin/streptomycin in a 37°C incubator with 5% CO_2_. The cells were passaged every 3 days at a confluence of 70–80% approximately.

### Reporter Vector Construction, Transfection, and Luciferase Assay

The putative promoter segment of 1 328 bps in the *ACE2* promoter region (chrX: 15 599 868—15 601 196; hg38) harboring rs2106809*A and rs2106809*G was directly synthesized by Sangon Biotech (Shanghai) Co., Ltd. The endonucleases HindIII and KpnI were involved in the putative promoter segment synthesis and cloned into the pGL3-Basic vector (Promega Ltd., E1754) to generate two luciferase vectors with rs2106809*A and rs2106809*G, respectively. The correctness of the constructs was verified by direct sequencing. The equal amounts of the two luciferase vectors were, respectively, transfected into A549 and HEK293T cells by Lipofectamine 3000 (Thermo Fisher Scientific Ltd., L3000015). A luciferase assay was performed according to the instruction of the Dual Luciferase Reporter Gene Assay Kit (YEASEN Biotechnology Co., Ltd., 11402ES60). The bioluminescence of firefly luciferase promoted by the putative promoter element of *ACE2* and of *Renilla* luciferase as the internal control was read by using a GloMax^®^ 20/20 Luminometer (Promega Ltd., E5311) at 560 and 480 nm, respectively. The final results were presented as the ratio between bioluminescence values of firefly luciferase and *Renilla* luciferase, as described before ^[22]^. The luciferase assay results were analyzed using the Student’s t-test, and the significance level was set at *p* < 0.05.

## Results

### Identification of the Putative Functional Single Nucleotide Polymorphism rs2106809 of *ACE2*

**FIGURE 1:**
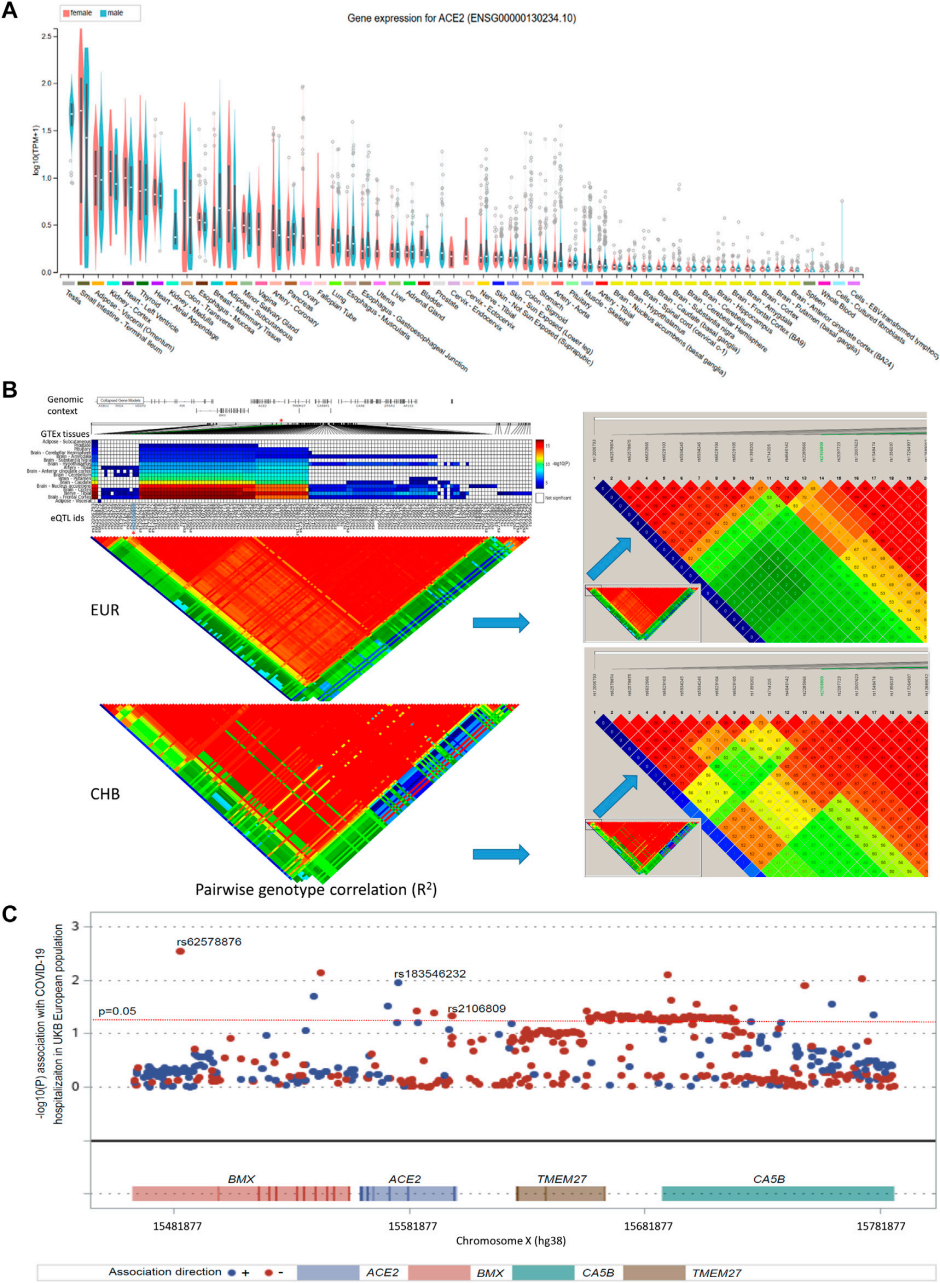
*ACE2* expression among multiple GTEx tissues **(A)**, comparison of the LD pattern of *ACE2* GTEx expression quantitative trait loci (eQTLs) (minor allele frequency > 0.05) between EUR and CHB populations **(B)**, and the association between rs2106809 with COVID-19 hospitalization in European population **(C)**. In panel A, the *ACE2* expression by sex among multiple GTEx tissues are demonstrated in violin plots. In adipose-subcutaneous and whole blood, the *ACE2* expression is higher in females than in males; while in breast mammary tissue, the *ACE2* expression is lower in females than in males (all *p* values < 0.01; ANOVA test). In panel B, there are 140 common eQTLs located in the genomic region of chrX: 15 284 068–16 013 888 (hg38). Only rs2106809 is a common SNP located in the promoter region of *ACE2* (chrX: 15 597 069–15 607 069; hg38), which is highlighted by “*”. rs2106809 displays the similar LD pattern with other GTEx eQTLs in both EUR and CHB populations. The pairwise genotype correlation between all GTEx eQTLs was determined using Haploview, and their corresponding *R* ^2^ values are shown with different colors, including red (R^2^ > 0.8), yellow (R^2^ between 0.5 and 0.8), blue (R^2^ between 0.2 and 0.5), and green (R^2^ < 0.2). In Panel C, rs2106809 along with two rare SNPs (rs62578876 and rs183546232) that are specific to EUR show an association with COVID-19 in the UK Biobank COVID-19 hospitalization GWAS of hospitalized cases (*n* = 1 712) vs. not hospitalized or tested negative samples (*n* = 56,988) in the EUR ancestry. rs2106809 minor allele G is protective against COVID-19 hospitalization (*p* = 0.047; beta = −0.07; se = 0.035), while the minor alleles of other two rare SNPs, including rs62578876 and rs183546232 (an intronic SNP of *ACE2*), are protective (*p* = 0.003; beta = −0.40; se = 0.13) and predisposing (*p* = 0.011; beta = 0.32; se = 0.12) to COVID-19 hospitalization, respectively. EUR: European; CHB: Chinese Han in Beijing.

**FIGURE 2:**
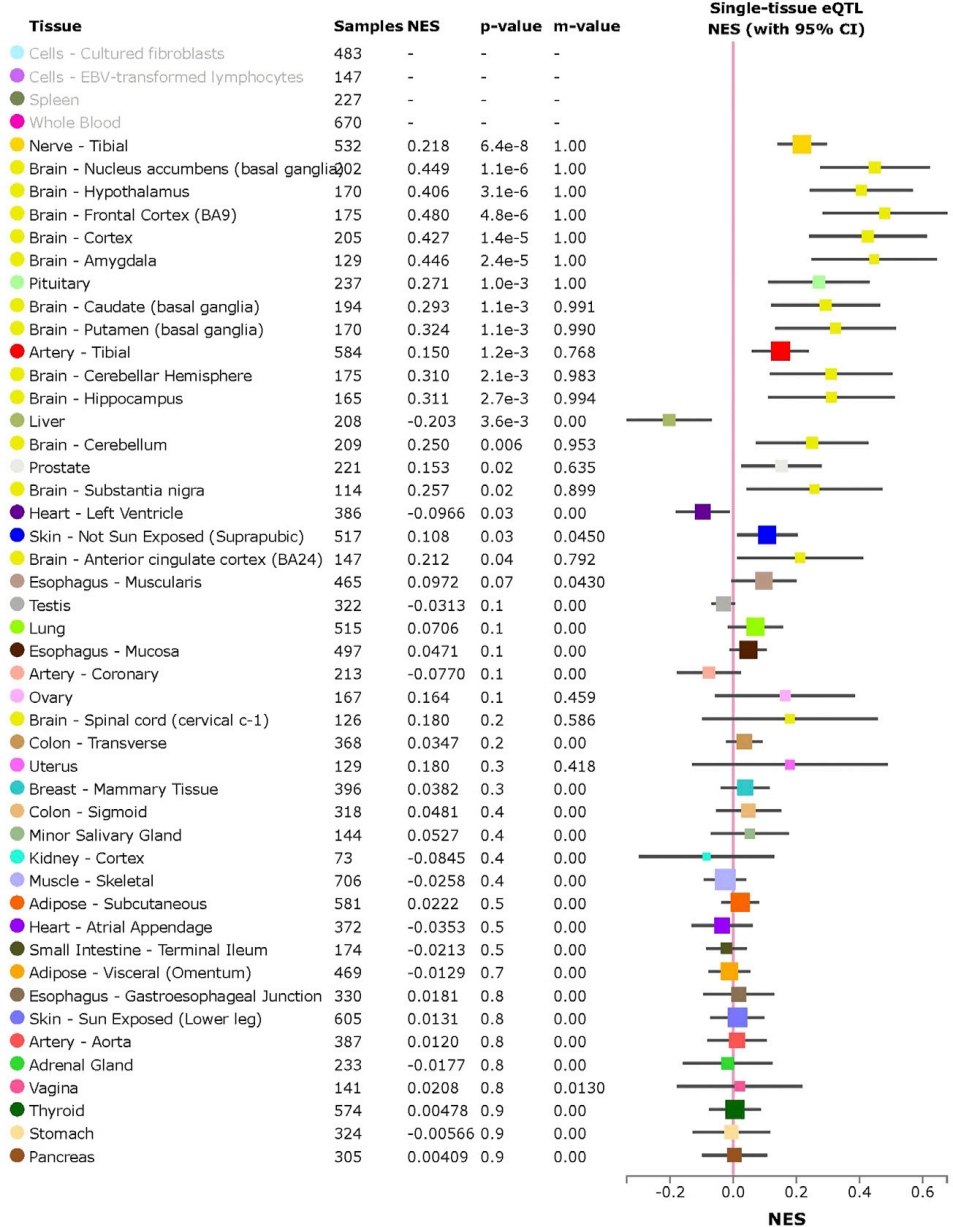
Single-tissue eQTL analysis of the *ACE2* promoter SNP rs2106809 across 49 human tissues in GTEx. rs2106809 is a strong eQTL of *ACE2* across multiple brain tissues, as well as in nerve and artery tissues. In lung tissue, rs2106809 is marginally associated with the *ACE2* expression (*p* = 0.07). NES: normalized effect size.

**FIGURE 3:**
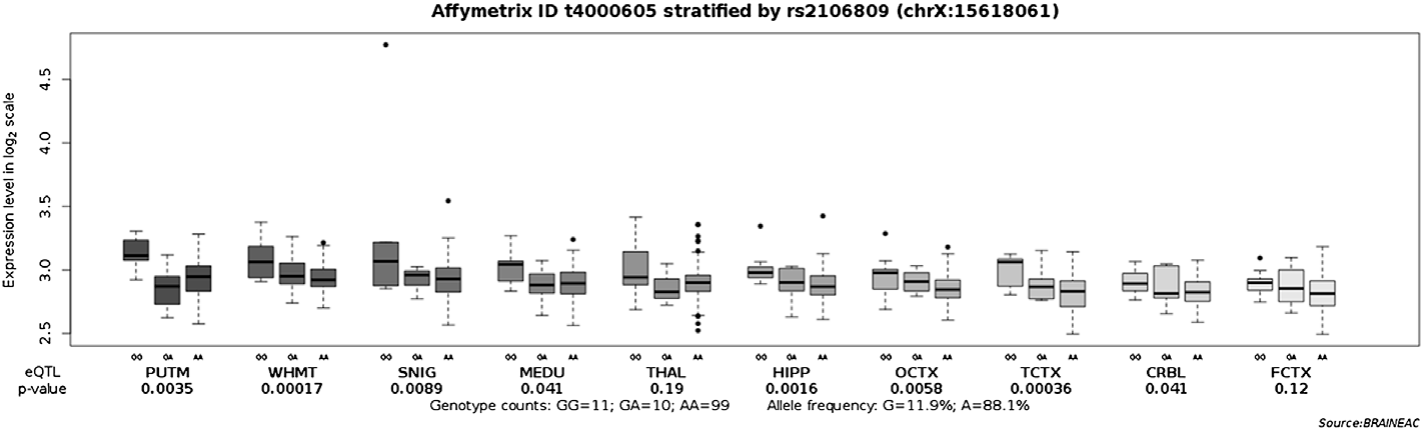
Replication of the associations of the *ACE2* promoter SNP rs2106809 with the *ACE2* expression across multiple brain tissues in the UK brain eQTL database, Braineac.

*ACE2* expression levels were determined across 49 human tissues *via* the GTEx database (Figure 1A). The testis, small intestine, adipose, kidney (cortex), and heart showed the highest *ACE2* expression levels. We also noticed that arterial and brain tissues presented higher *ACE2* expression levels than other tissues. LD analysis of *ACE2* eQTLs in EUR and CHB populations showed similar LD patterns (Figure 1B). In addition, the minor allele G of rs2106809 is a protective allele associated with COVID-19 hospitalization in the European population (*p* = 0.047; Figure 1C). The normalized effect size (NES) of the single-tissue eQTLs of the *ACE2* SNP rs2106809 across 49 human tissues in the GTEx database (Figure 2) revealed that most brain tissues display a correlation between *ACE2* expression and rs2106809. This association was replicated in the UK brain eQTL database, Braineac (Figure 3). Taken together, the promoter SNP rs2106809*G is tightly associated with a higher *ACE2* expression in multiple brain tissues, which also shows protective association with COVID-19 hospitalization in European population.

### rs2106809 G Allele Increasing the Promoter Activity in Luciferase Assay

**FIGURE 4:**
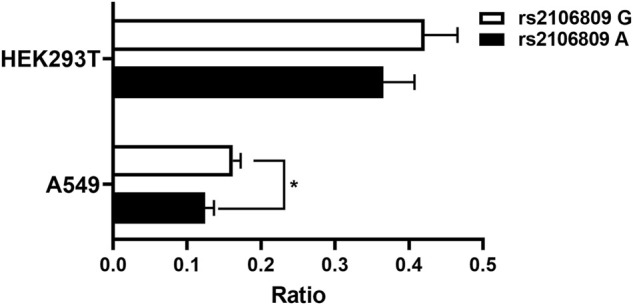
Luciferase assay for the putative promoter of *ACE2* in HEK293T and A549 cells. Two constructs representing rs2106809 genotype GG and genotype AA harbored in the promoter region (∼1.33kb; chrX: 15 599 868–15 601 196; hg38) were, respectively, inserted into the pGL3-Basic vector and then transfected into HEK293T and A549 cells separately. A *Renilla* luciferase vector was co-transfected as an internal control. The luciferase activity was measured at 24 h post transfection. The ratio of firefly to *Renilla* bioluminescence is presented as the mean + SD of triplicated transfection in one representative experiment (*: *p* < 0.05).

Based on genetic analysis, we predicted rs2106809 would affect the promoter activity of *ACE2*. In order to verify such prediction, we separately cloned two luciferase vectors with the genotype GG and AA of rs2106809 into the pGL3-Basic vector and confirmed it by sequencing (Supplementary Figure S1). The two luciferase vectors were, respectively, transfected into HEK293T cells and A549 cells for luciferase activity evaluation. The dual luciferase-reporter gene assay showed rs2106809 GG increased the promoter activity than the AA genotype in HEK293T cells and significantly enhanced the promoter activity than the AA genotype in A549 cells (Figure 4, *p* < 0.05). The results of luciferase assay suggested that rs2106809 was closely correlated with the differential *ACE2* expression.

## Discussion

We mapped the eQTLs of *ACE2* that existed in the *ACE2* promoter in EUR and CHB populations and verified the possible regulatory function of one candidate SNP rs2106809, which is a brain eQTL of *ACE2*. In our detailed examination of the COVID-19 hospitalization GWAS of the EUR ancestry from the UK Biobank (Figure 1C), we revealed the minor allele G of rs2106809 is protective against COVID-19 hospitalization, although its association was only nominally significant (*p* = 0.047). A recent epidemiological investigation from China demonstrated that among COVID-19 cases, there were 37.6% hospitalized COVID-19 patients diagnosed with long COVID ^[23]^, and another study from Faroe Islands, part of the Kingdom of Denmark, reported that around 50% of non-hospitalized COVID-19 patients developed into long COVID ^[24]^. We hypothesized that severe COVID-19 and long COVID are two different phenotypes; given the high frequency of the rs2106809 major allele A in EUR and CHB populations, further investigation is needed to determine whether the A allele of rs2106809 is associated with long COVID symptoms, such as “brain fog”.

There were several interesting scientific literature reports focused on the *ACE2* SNP rs2106809. A multicentral clinical trial of 3,408 patients found that the rs2106809 major allele A conferred a 1.6-fold risk for hypertension in Chinese women ^[25]^. The subsequent research that enrolled 647 Chinese Han patients concluded that the rs2106809 A allele was statistically associated with left ventricular hypertrophy with 2.0-fold risk ^[26]^. The other report showed a remarkable relationship between the rs2106809 polymorphism and essential hypertension (EH) in 246 hypertensives and 274 normotensives from Odisha, India ^[27]^. Notably, a clinical survey of 96 Chinese female EH patients found the circulating Ang (1–7) levels were significantly higher in patients carrying the rs2106809 G allele than those carrying the A allele ^[28]^, in line with the fact that rs2106809*G associates with a higher *ACE2* expression, and a higher ACE2 activity increases the amount of Ang ^[6]^. Such a series of epidemiological investigations provided various solid pieces of evidence that the *ACE2* SNP rs2106809 possessed a tight relationship with EH in Chinese and Indian populations. The A allele preferred to confer a higher risk for EH and EH-related cardiovascular disease. On the contrary, the G allele preferred to confer ameliorated EH. However, a case study of 155 COVID-19 patients of Turkey demonstrated the *ACE2* rs2106809 polymorphism was not associated with the clinical severity of COVID-19 infection ^[29]^. In our analysis of the European COVID-19 hospitalization GWAS performed among all SARS-CoV-2 tested samples (cases = 1 712 and controls = 56,988), rs2106809*G is protective against COVID-19 hospitalization. Since ACE2 is a cellular receptor of SARS-CoV-2 and is also involved in hypertension that delays viral clearance and exacerbates airway hyperinflammation in patients with COVID-19 ^[30]^, this conclusion may need to be verified with more COVID-19 cases and among multiple human populations, such as CHB, EUR, and Yoruba in Ibadan, Nigeria.

There are several limitations that should be mentioned. First, our current study did not present an epidemiological investigation on the relationship between severe COVID-19 and long COVID in CHB, which needs to be investigated further. Second, our data did not include the COVID-19 or long COVID patients who carry this SNP; thus, we could not examine the association between the SNP with the two COVID-19 phenotypes in CHB. Third, the 1,328-bp promoter region of *ACE2* used in our study was synthesized based on reference sequence from GenBank (chrX: 15 599 868—15 601 196; hg38); however, such a segment from real samples of patients with EH, severe COVID-19, or long COVID, might offer more valuable information to this study. Fourth, the cells used in luciferase assay did not include the astrocyte cell line such as HMC3 ^[31]^. The result of the dual luciferase reporter gene assay could not fully represent the real *ACE2* expression across different tissues. Finally, more SNPs of *ACE2*, particularly for population-specific rare SNPs, should be assessed to find out whether they are associated with severe COVID-19 or long COVID.

Despite these limitations, our findings provide solid information on the correlation between rs2106809*G and the remarkably higher expression level of *ACE2* in cerebral tissue, the site where brain fog was characterized in long COVID. We integrated the data from GTEx and Braineac to interpret the potential function of rs2106809 to the *ACE2* expression. Importantly, we experimentally confirmed rs2106809 regulates the *ACE2* expression. In conclusion, our results are based on solid evidence *via* bioinformatics analyses and the dual luciferase reporter gene assay that demonstrated the rs2106809 G allele was able to significantly increase the expression of *ACE2*. The identification of rs2106809 as an *ACE2* eQTL among multiple brain tissues supports that rs2106809 may be involved in long COVID, but further investigations are warranted.

## Data Availability

The datasets presented in this study can be found in online repositories. The names of the repository/repositories and accession number(s) can be found in the article/Supplementary Material.
