# Supplementary figures and images for: Evaluation of a Functional Single Nucleotide Polymorphism of the SARS-CoV-2 Receptor ACE2 That Is Potentially Involved in Long COVID
Source: Front Genet. 2022 Jul 18;13:931562. doi: 10.3389/fgene.2022.931562 (PMC9340221; doi:10.3389/fgene.2022.931562)

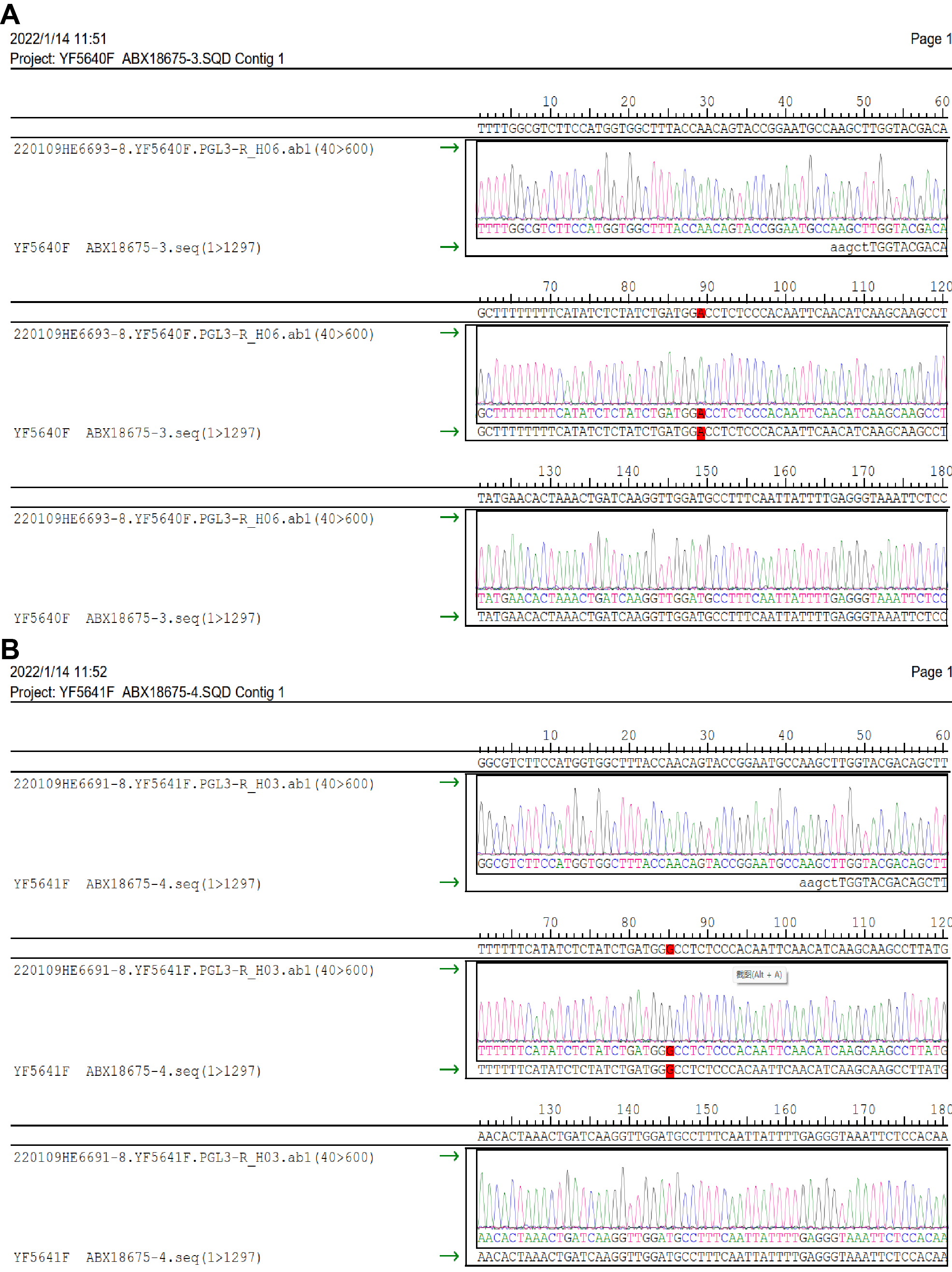

Supplement: Supplementary file 1 [file Image1.TIF]
